# Isolated Subclinical Right Ventricle Systolic Dysfunction in Patients after Liver Transplantation

**DOI:** 10.3390/jcm12062289

**Published:** 2023-03-15

**Authors:** Emel Celiker Guler, Mehmet Onur Omaygenc, Deniz Dilan Naki, Arzu Yazar, Ibrahim Oguz Karaca, Esin Korkut

**Affiliations:** 1 Department of Cardiology, Koc University Hospital, Davutpasa Ave., No:4, Zeytinburnu, 34010 Istanbul, Turkey; 2 Department of Cardiology, Istanbul Medipol University Hospital, TEM European Highway, Goztepe Exit, No:1, Bagcilar, 34214 Istanbul, Turkey

**Keywords:** end-stage chronic liver disease, liver transplantation, heart failure, right ventricle function, propranolol

## Abstract

Although hemodynamic alterations in end-stage liver disease (ESLD) and its association with porto-pulmonary hypertension have been well-established, the long-term effects of ESLD on RV systolic function in patients without porto-pulmonary hypertension remain disregarded. Here we aimed to assess the long-term effect of ESLD on RV function and its relationship with the use of NSBBs and clinical, laboratory and imaging parameters in end-stage liver disease. The use of NSBBs is still controversial due to concerns about reduced cardiac contractility and the possibility of increased mortality. Thirty-four liver transplant recipients were included. Demographic characteristics, laboratory and baseline echocardiography measures were obtained. Patients were recalled for transthoracic echocardiographic evaluation after transplantation. Right ventricle dysfunction was identified by having at least one value below the reference levels of RV S’, or TAPSE. Isolated subclinical RV dysfunction was observed at 20.6% of the sample population. The present study demonstrates hemodynamic circulation in cirrhosis and increased preload and afterload might have long-term effects on RV function, even the lack of porto-pulmonary hypertension. These findings underline the significance of cardiac function follow-up in cirrhotic patients after transplantation. In this study, patients treated with propranolol seemed to have better RV function and less gastrointestinal bleeding. We speculated that preoperative propranolol treatment might help preserve RV function by providing RAS suppression, improving endothelial function and hyperdynamic circulation seen in ESLD. This potential protective relationship between the use of propranolol and RV function might improve mortality or graft-failure during OLT and after liver transplantation in patients with cirrhosis.

## 1. Introduction

End-stage liver disease (ESLD) affects the pulmonary and cardiac structure and function through several mechanisms. Although the exact mechanism is not clear yet, several hypotheses including hyperdynamic circulation, excess production and impaired clearance of vasoactive substances, portosystemic shunt and genetic predisposition are considered to have a role in this multisystemic effect [1,2,3]. Hyperdynamic circulation refers to increased cardiac output (CO), reduced systemic vascular resistance and splanchnic vasodilation noted in patients with cirrhosis [4,5]. High CO may lead to endothelial dysfunction due to shear wall stress. Moreover, toxic substances that bypass the liver through the portosystemic shunt may also cause direct damage to the pulmonary arteriolar endothelium [3,4,6]. These multifactorial mechanisms may result in excessive pulmonary vascular remodeling, increased pulmonary vascular pressures and pulmonary vascular resistance (PVR), and consequently, right ventricular (RV) dysfunction [7]. Although more commonly considered in the context of pulmonary hypertension, cirrhotic cardiomyopathy also induces left ventricle (LV) dysfunction and rising RV afterload owing to increased LV diastolic pressures. Severely increased RV afterload is recognized as a risk factor for mortality in liver transplant candidates. The deterioration of RV function has been demonstrated to be superior to LV dysfunction in predicting adverse clinical outcomes and mortality during orthotopic liver transplantation (OLT) [8].

Non-selective beta blockers (NSBBs) are recommended for the primary and secondary prevention of variceal bleeding in patients with cirrhosis [9,10,11,12,13]; their protective effect on recurrent gastrointestinal bleeding was first introduced in the literature almost 40 years ago [13]. Non-selective beta blockers reduce portal hypertension by reducing portal venous flow through splanchnic vasoconstriction. Moreover, NSBBs might decrease systemic inflammation via increased intestinal transit and reduced gut permeability [14]. Many studies have shown that NSBBs are associated with improved survival in patients with cirrhosis [15,16]; however, the use of NSBBs in ESLD is still controversial due to their harmful effects on cardiac contractility and mean arterial pressure [17,18]. However, compared to NSBBs, selective beta-1 antagonists such as atenolol and metoprolol have been shown to be less effective for the prevention of variceal bleeding [19,20]. Non-selective beta blockers decrease portal pressure by beta-2 adrenergic blockage, which causes splanchnic vasoconstriction; however, selective beta-1 antagonists have no effect on portal pressure. A prospective randomized trial assessed for the value of selective beta-1 antagonists for the long-term management of variceal bleeding showed that metoprolol was associated with a significantly higher risk of recurrent variceal bleeding compared to injection sclerotherapy [19].

Two-dimensional (2D) transthoracic echocardiography is a routine examination for the assessment of porto-pulmonary hypertension [21], RV/LV structure and function [22,23], porto-pulmonary shunt [24] and the necessity of right heart catheterization among patients with ESLD being considered for liver transplantation. The association of RV functional impairment and advanced chronic liver disease in patients with porto-pulmonary hypertension has been well-established. However, the long-term effects of ESLD and increased RV afterload in RV systolic function remain disregarded especially in cirrhotic patients without signs of porto-pulmonary hypertension. Therefore, we sought to determine whether ESLD would have long-term effects on RV function and cause subclinical RV systolic dysfunction without the clinical/echocardiographic signs of porto-pulmonary hypertension. Finally, we sought to determine the impact of non-selective beta blockers and other identifiers on RV systolic function after OLT by comparing the baseline characteristics and follow-up echocardiographic parameters.

## 2. Materials and Methods

### 2.1. Study Population

The analysis was performed on consecutive adult ESLD patients (age > 18 years) who underwent their first liver transplantation at Medipol University from June 2014 to September 2019. At our institution, echocardiography is a standard of care for all OLT candidates. Orthotopic liver transplantation candidates who had significant left heart disease, severe chronic pulmonary disease, any identified previous episode of pulmonary embolism, hepatocellular carcinoma as the etiology for transplant, <6 months between transplant and echocardiographic follow-up or the absence of critical retrospective data were excluded. Demographic and baseline clinical features at the time of surgery were noted. Preoperative laboratory work-up, estimated systolic pulmonary arterial pressure (sPAP) value at preoperative cardiology consultation and post-transplantation early hepatic and portal venous duplex ultrasound findings were recorded. Patients were recalled for follow-up echocardiographic examination.

The study was approved by the Institutional Ethics Review Committee of Medipol University (299/20 and 16 April 2020).

### 2.2. Echocardiography

Echocardiography was performed using a commercially available echocardiography system (Vivid 7 or E9 (General Electric-Vingmed, Horten, Norway)) and the obtained images were digitally stored in cine-loop format. The EchoPAC system was used for offline analysis (EchoPAC version 112.0.1; General Electric-Vingmed Ultrasound, Horten, Norway). The standard biplane Simpson method was used to calculate the left ventricular ejection fraction (LVEF) [13]. Pulsed-Doppler echocardiography was performed to measure peak early diastolic transmitral flow velocity (E) [13]. The early diastolic velocity of the lateral and septal aspects of the mitral annulus (E’) was measured by Doppler tissue imaging and was averaged to acquire E’. The E/E’ ratio was calculated by dividing the peak inflow velocity by the averaged annular velocities [23]. The maximum tricuspid regurgitant jet gradient was calculated by using the modified Bernoulli equation [14]. Systolic pulmonary arterial pressure was calculated by summating the tricuspid regurgitant gradient and right atrial pressure. Tricuspid annular plane systolic excursion (TAPSE) was measured from M-mode recordings of the lateral tricuspid annulus in the RV view [25]. Measurements of tricuspid annular systolic velocity (TAs) were obtained using tissue Doppler imaging [14].

### 2.3. Statistical Analysis

The data were presented as mean ± standard deviation and median (range) for continuous variables and percentage for categorical variables. Shapiro–Wilk test was used to determine whether a continuous variable followed normal distribution or not. To discriminate the patients with and without right ventricular dysfunction, Student’s t and Mann–Whitney U tests were utilized for comparing the variables showing normal and non-normal distribution, respectively. The frequencies of categorical variables were distinguished by the chi-square test. Two-sided *p* ≤ 0.05 was interpreted as statistically significant. Statistical analyses were carried out by using SPSS (version 17.0, SPSS Inc., Chicago, IL, USA).

## 3. Results

A total of 56 liver transplant recipients who had the complete baseline dataset were assessed for recruitment. Seven patients were excluded due to confounding medical conditions as described above (exclusion criteria: OLT candidates who had significant left heart disease, severe chronic pulmonary disease, any identified previous episode of pulmonary embolism, hepatocellular carcinoma as the etiology for transplant, <6 months between transplant and echocardiographic follow-up or the absence of critical retrospective data). Of the remaining 49 recipients, 15 individuals were lost to follow-up.

Ultimately, 34 patients (mean age 54.1 ± 8.7) constituted the sample population. Follow-up echocardiography was performed after a mean duration of 18 ± 6 months following OLT. At the follow-up visit, right ventricular dysfunction (RVD) was identified as the measurement of TAPSE and/or peak Doppler annular velocity (RVSm) below the reference values (<17 mm and 9.5 cm/s, respectively) suggested in the relevant position paper [13]. Isolated RV dysfunction was observed at 20.6% (*n* = 7) of the sample population. Patients were then grouped according to the presence of isolated RV dysfunction (Group A: liver recipients without isolated subclinical RV dysfunction and Group B: liver recipients with subclinical RV dysfunction)

Table 1 provides the baseline characteristics of the patients with isolated RV dysfunction and without RV dysfunction. The age, gender and body mass index (BMI) were similar between the two groups. The mean time between diagnosis and transplant was 5.5 ± 5.3 years and was similar in the two groups. Viral etiology hepatitis was seen in approximately half of the patients in both groups. Hepatocellular carcinoma as the etiology for transplant was excluded. Other etiologies including alcoholic liver disease and non-alcoholic fatty liver diseases were similar between the two groups. There were no statistically significant differences in the Child–Pugh score, Model for End-stage Liver Disease (MELD) score and esophageal variceal grade between the two groups; however, gastrointestinal bleeding was higher in group B (Table 1). The history of hypertension and smoking between the two groups showed no significant difference. However, the history of coronary artery disease (CAD) was higher in group B. Treatment with spironolactone, other diuretics, renin-angiotensin system blocking agents (RAS-B) and calcium channel blockers (CCB) was similar in both groups. In contrast, treatment with a non-selective beta blocker (propranolol) was higher in group A (Table 1).

**Figure 1:**
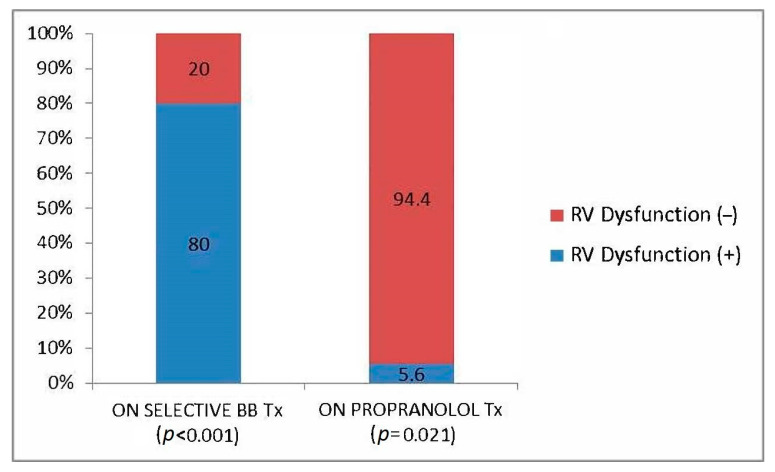
Relationship of non-selective beta blocker and isolated subclinical RVD.

The choice of non-selective beta blocker was propranolol, as nadolol is non-available in Turkey. Patients who used selective beta-blocking agents due to coronary artery disease and hypertension and did not use propranolol seemed to have higher rates of isolated RV dysfunction (RVD) (Figure 1).

Preoperative laboratory test variables between the two groups including estimated glomerular filtration rate (eGFR), albumin, INR, ALT, GGT, hemoglobin, platelet count, CRP and pro-BNP were similar (Table 2). In addition, preoperative hepatic and portal venous duplex US findings were similar between the two groups (Table 2).

A comparison of echocardiographic parameters in the two groups is shown in Table 3. In both groups, preoperative and follow-up sPAP values were within the normal range. There was no statistical difference between the two groups in LV Ejection Fraction (LVEF), E/E’, LV/RV ratio and RA area.

## 4. Discussion

In this study, we demonstrated that ESLD has long-term effects on RV function. To the best of our knowledge, this is the first study describing the term “Isolated subclinical right ventricular dysfunction” after orthotopic liver Tx. It is noted that increased preload and afterload in ESLD have an impact on RV function even in the absence of porto-pulmonary hypertension. This study underlines the importance of the long-term follow-up of cardiac functions in patients with cirrhosis. In addition, we also noted that preoperative treatment with propranolol might help preserve RV function. There might be a potential protective relationship between the use of propranolol and RV function in patients with cirrhosis. This finding can be explained by the beneficial effects of PPL in endothelial function, RAS suppression and hyperdynamic circulation that might promote RV function during long-term follow-up in patients with ESLD. Moreover, the patients treated with non-selective beta-blockers had less gastrointestinal bleeding than those who did not use propranolol. This finding agrees with the previous studies, as propranolol is considered effective in the primary and secondary prevention of variceal bleeding.

The clinical importance of subclinical RV dysfunction and its association with adverse clinical outcomes has been investigated in different clinical scenarios [26,27,28,29,30,31]. In ESLD, RV afterload and preload are affected due to several hemodynamical consequences of advanced liver disease including hyperdynamic circulation, increased pulmonary vascular resistance and increased LV diastolic pressure with a subsequent increase in pulmonary wedge pressure [3,4,5,6,7]. Given the sensitivity of RV to dynamic alterations in preload and afterload, these multifactorial mechanisms in advanced liver disease can be a concern in the development of RV overload and dysfunction. Demirtas Inci et al. compared the global LV and RV functions of forty liver transplant candidates with ESLD and twenty-six healthy individuals [32]. The 2D speckle tracking method was preferred for the mean longitudinal, circumferential and radial strain measurements. They noted that LV mean radial/longitudinal and RV mean longitudinal strain were significantly lower in the patient group. The study concluded that the deterioration of longitudinal and radial deformation is a sign of the subclinical impairment of global LV and RV systolic functions in liver transplant candidates [32].

Other studies have evaluated RV function in ESLD patients while awaiting and during transplants [22,33,34]. A retrospective study compared TAPSE and TAs between a group of ESLD patients being considered for OLT and patients without the liver disease [22]. All patients included in the study had normal LV systolic function and normal pulmonary artery systolic pressures [22]. They found that TAPSE and TAs values were significantly higher in patients with ESLD awaiting liver transplantation [34]. The authors suggested the differences in echocardiographic indices of RV systolic function led to a hyperdynamic circulatory state in ESLD patients [34]. A limitation of this study is the unknown long-term impact of this compensatory mechanism on RV function following transplantation. Right ventricle function during classic OLT has also been investigated [34]. Xu et al. examined RVEF using a modified pulmonary artery catheter during OLT in a study of thirty patients [34]. Baseline RVEF was found to be lower than the normal value. In addition, Xu et al. noted that RVEF was impaired during the anhepatic and early reperfusion stages. The authors highlighted that the RV function plays a crucial role in maintaining stable hemodynamics and close monitoring of the RV function is essential [34]. Moreover, a study has shown that right heart-associated hemodynamic factors were associated with patient survival after liver transplantation or graft failure [35]. Even though no association between the time-weighted LV stroke work index and PVR for one-year mortality was demonstrated, the time-weighted mean RV stroke work index was found significantly related with one-year all-cause mortality. The study advised that the intraoperative RV stroke work index might be a prognostic marker for mortality after liver transplantation [35]. In our study, patients treated with NSBBs had significantly less RV systolic dysfunction, which might be associated with good outcome in this population both during transplantation and follow-up.

Non-selective beta blocker agents, mostly propranolol (PPL), are used for the primary and secondary prophylaxis of acute and chronic gastrointestinal (GI) bleeding caused by esophageal varices in patients with cirrhosis. Carvedilol, nebivolol and nadolol are alternative agents [36,37]. Propranolol decreases portal hypertension and portal blood flow by reducing CO and splanchnic vasoconstriction [38,39]. In our study, the preferred non-selective beta blocker was propranolol. Indeed, carvedilol would be the preferred B blocker agent because, in recent years, several studies demonstrated that it was better in long-term HVPG reduction due to its anti-alpha-adrenergic activity and ability to improve the release of nitric oxide. Furthermore, a recent study demonstrated that patients treated with carvedilol had better adherence to therapy and outcome than propranolol [40]. However, most of these findings were obtained after this study was designed. Additionally, nadolol was not preferred as it is non-available in our country. Our study found that the patients treated with propranolol had less gastrointestinal bleeding.

Besides the beneficial effects of propranolol in reducing hepatic vein pressure gradient (HVPG), it has been reported in some studies that propranolol might alleviate inflammatory response, reduce bacterial translocation, improve endothelial function, reduce the angiotensin levels in the portal vein and peripheral vasculature [41,42,43,44]. A prospective controlled study investigated PPL’s vascular function and impact using venous occlusion plethysmography [43]. The authors demonstrated the gradual deterioration of endothelial function in cirrhotic patients not treated with PPL; nevertheless, there was an improvement in Child–Pugh score in patients treated with PPL [43]. These findings also supported another study that included 38 cirrhotic outpatients with ascites, divided into two groups according to use or not of PPL [41]. They demonstrated that PPL might protect the endothelium from inflammatory exhaustion and improve hemodynamic status [41]. Furthermore, Vilas-Boas et al. found that treatment with PPL reduced the Renin–Angiotensin System (RAS) mediators and could change the prognosis of patients with hyperdynamic circulation [43]. In the present study, we demonstrated that the patients treated with PPL had significantly lower rates of isolated RV dysfunction compared to those not treated with PPL. The reduced RAS activation and decreased hyperdynamic circulation, in addition to a less inflammatory response with PPL, might be protective of RV function during long-term follow-up.

On the contrary, a study investigated increased mortality with NSBBs in patients with refractory ascites and cirrhosis [17]. This single-center study evaluated 1-year survival at 19% in patients treated with propranolol; however, 1 year survival of 64% in patients not treated with propranolol suggested that the use of propranolol in patients with cirrhosis and refractory ascites was associated with poor survival [17]. Furthermore, ‘the window therapy hypotheses’ were proposed by Krag et al., where NSBBs would be used in the early stages without severe varices and should be stopped in end-stage cirrhosis because they would reduce cardiac contractility, worsening systemic perfusion and prognosis [18]. In advanced cirrhosis, compensatory mechanisms including up-regulation of the sympathetic nervous system [45,46] and renin-angiotensin-aldosterone system [47,48] exist to maintain proper cardiac output and organ perfusion; however, these compensatory mechanisms fail as cirrhosis progresses. In this advanced stage, the maintenance of cardiac output and mean arterial pressure is essential to improve survival. However, recent studies and the BANEVO VII guidelines recommended the use of NSBBs in all patients with clinically significant portal hypertension [49]. The PREDESCI trial, a multicenter, double-blind and first randomized controlled trial, showed that long-term treatment with NSBBs reduces clinical decompensation or cirrhosis-related mortality by reducing the rates of ascites. The study suggested that the long-term treatment of cirrhotic patients with NSBBs might prevent progression to clinical decompensation or death [40].

Some limitations should be acknowledged, such as the single-center nature of the study, the selection biases of a retrospective study and sample size. Moreover, only 2D echocardiographic parameters were assessed for the evaluation of RV function. Strain imaging and novel 3-dimensional echocardiography are preferred to support the present study’s findings.

## 5. Conclusions

To the best of our knowledge, this is the first study describing the term “Isolated subclinical right ventricular dysfunction” after orthotopic liver Tx. We hypothesized that the impact of propranolol on RAS suppression, hemodynamic circulation and endothelial function might help preserve RV function during long-term follow-up via a more prominent reduction in hepatic venous gradient at the preoperative stage. This potential protective relationship between the use of propranolol and RV function in patients with cirrhosis might improve mortality or graft-failure after liver transplantation. However, future studies with more robust designs are necessary to verify this association and the hypothesis should be supported by evidence obtained via biochemical work-up.

## Figures and Tables

**Table 1:** Baseline characteristics of the liver transplant recipients.

	Overall (*n* = 34)	RVD (−) (*n* = 27)	RVD (+) (*n* = 7)	*p* Value
Age, years; Mean ± SD	54.1 ± 8.7	53.6 ± 8.7	56.1 ± 9.1	0.65
Gender, female; % (*n*)	32.4 (11)	29.6 (8)	42.9 (3)	0.51
BMI, kg/m^2^; Median (Range)	29.6 [11.5]	29.4 [11.5]	30.5 [7.4]	0.40
Diagnosis-to-transplantation time, years; Mean ± SD	5.5 ± 5.3	5.8 ± 5.5	4.6 ± 4.3	0.71
Etiology, viral; % (*n*)	47.1 (16)	48.1 (13)	42.9 (3)	0.80
Child–Pugh score; Mean ± SD	8.2 ± 1.6	8.3 ± 1.5	8.5 ± 2.6	0.43
MELD score; Mean ± SD	18.3 ± 6.3	19.3 ± 6.3	14.6 ± 5.1	0.06
Esophageal varices grade; Mean ± SD	1.4 ± 1.1	1.5 ± 1.1	1.3 ± 1.1	0.65
History of GI Bleeding; % (*n*)	17.6 (6)	11.1 (3)	42.9 (3)	**0.05**
Hypertension; % (*n*)	38.2 (13)	44.4 (12)	14.3 (1)	0.14
Smoking history (>20 package years); % (*n*)	41.2 (14)	37 (10)	57.1 (4)	0.34
Coronary artery disease; % (*n*)	5.9 (2)	0 (0)	28.6 (2)	**<0.01**
Propranolol use; % (*n*)	52.9 (18)	63 (17)	14.3 (1)	**0.02**
Spironolactone use; % (*n*)	61.8 (21)	63 (17)	57.1 (4)	0.78
Other diuretic use; % (*n*)	67.6 (23)	66.7 (18)	71.4 (5)	0.81
RAS-B use; % (*n*)	14.7 (5)	18.5 (5)	0 (0)	0.22
CCB use; % (*n*)	17.6 (6)	18.5 (5)	14.3 (1)	0.79

**Table 2:** Preoperative laboratory test results and hepatic and portal venous duplex US findings of the liver transplant recipients.

	RVD (−) (*n* = 27)	RVD (+) (*n* = 7)	*p* Value
eGFR, mL/min/1.73 m^2^; Median (Range)	99.7 [143.5]	99.8 [85.2]	0.93
Albumin, g/dL; Mean ± SD	3.3 ± 0.6	3.3 ± 0.6	0.97
INR, Median (Range)	1.5 [2.1]	1.5 [1]	0.93
ALT, U/L; Mean ± SD	42 ± 31.1	39.5 ± 26.7	0.87
GGT, U/L; Mean ± SD	69 ± 62.1	131 ± 119.5	0.16
Hemoglobin, g/dL; Median (Range)	10.5 [9.2]	12.6 [5]	0.24
Platelet count, ×1000/µL; Mean ± SD	87.8 ± 55.8	148.1 ± 152.9	0.59
CRP, mg/L; Mean ±SD	16.3 ± 24.5	14 ± 18.1	0.90
NT-ProBNP, pg/mL; Mean ± SD	161 ± 166.5	257.9 ± 353.8	0.30
Portal vein diameter, mm; Median (Range)	10 [7.2]	11 [3.8]	0.59
Portal vein velocity, cm/s; Mean ± SD	113.4 ± 77.2	72.3 ± 45.4	0.18
Portal vein output, mL/min; Mean ± SD	2460 ± 1787	2122 ± 1125	0.87
Hepatic vein velocity, cm/s; Mean ± SD	59.7 ± 32.4	56 ± 36	0.62

**Table 3:** Preoperative and follow-up echocardiography parameters in liver transplant recipients.

	RVD (−) (*n* = 27)	RVD (+) (*n* = 7)	*p* Value
Initial estimated sPAP, mmHg; Mean ± SD	29.6 ± 6	28.3 ± 2.2	0.68
Follow-up estimated sPAP, mmHg; Mean ± SD	28 ± 7.5	27.6 ± 7	0.87
LVEF, %; Mean ± SD	64 ± 3.3	63.3 ± 2.6	0.29
E/E’; Mean ± SD	7.2 ± 1.9	7 ± 2.2	0.84
RV:LV ratio; Median (Range)	0.75 [0.28]	0.77 [0.35]	0.36
RA area, cm^2^; Median (Range)	12.5 [11.6]	14.4 [7]	0.45

## Data Availability

The data presented in this study are available on request from the corresponding author. The data are not publicly available due to privacy restrictions.

## References

[1] Battista S., Bar F., Mengozzi G., Zanon E., Grosso M., Molino G. (1997). Hyperdynamic circulation in patients with cirrhosis: Direct measurement of nitric oxide levels in hepatic and portal veins. J. Hepatol..

[2] Savale L., Watherald J., Sitbon O. (2017). Portopulmonary Hypertension. Semin. Respir. Crit. Care Med..

[3] Liberal R., Grant C.R., Baptista R., Macedo G. (2015). Porto-pulmonary hypertension: A comprehensive review. Clin. Res. Hepatol. Gastroenterol..

[4] Herve P., Lebrec D., Brenot F., Simonneau G., Humbert M., Sitbon O., Duroux P. (1998). Pulmonary vascular disorders in portal hypertension. Eur. Respir. J..

[5] Rodríguez-Vilarrupla A., Fernández M., Bosch J., García-Pagán J.C. (2007). Current concepts on the pathophysiology of portal hypertension. Ann. Hepatol..

[6] Herve P., Le Pavec J., Sztrymf B., Decante B., Savale L., Sitbon O. (2007). Pulmonary vascular abnormalities in cirrhosis. Best Pract. Res. Clin. Gastroenterol..

[7] Wong F., Liu P., Lilly L., Bomzon A., Blendis L. (1999). Role of cardiac structural and functional abnormalities in the pathogenesis of hyperdynamic circulation and renal sodium retention in cirrhosis. Clin. Sci..

[8] Kia L., Shah S.J., Wang E., Sharma D., Selvaraj S., Medina C., Cahan J., Mahon H., Levitsky J. (2013). Role of pretransplant echocardiographic evaluation in predicting outcomes following liver transplantation. Am. J. Transplant..

[9] Jakab S.S., Garcia-Tsao G. (2020). Evaluation and management of esophageal and gastric varices in patients with cirrhosis. Clin. Liver. Dis..

[10] Poynard T., Cales P., Pasta L., Ideo G., Pascal J.P., Pagliaro L., Lebrec D., Franco—Italian Multicenter Study Group (1991). Beta-adrenergic-antagonist drugs in the prevention of gastrointestinal bleeding in patients with cirrhosis and esophageal varices: An analysis of data and prognostic factors in 589 patients from four randomized clinical trials. N. Engl. J. Med..

[11] Pascal J.P., Cales P. (1987). Propranolol in the prevention of first upper gastrointestinal tract hemorrhage in patients with cirrhosis of the liver and esophageal varices. N. Engl. J. Med..

[12] Garcia-Tsao G., Bosch J. (2010). Management of varices and variceal hemorrhage in cirrhosis. N. Engl. J. Med..

[13] Lebrec D., Corbic M., Nouel O., Benhamou J.P. (1980). Propranolol—A medical treatment for portal hypertension?. Lancet.

[14] Senzolo M., Cholongitas E., Burra P., Leandro G., Thalheimer U., Patch D., Burroughs A.K. (2009). β-Blockers protect against spontaneous bacterial peritonitis in cirrhotic patients: A meta-analysis. Liver Int..

[15] Lo G.-H., Chen W.-C., Lin C.-K., Tsai W.-L., Chan H.-H., Chen T.-A., Yu H.-C., Hsu P.-I., Lai K.-H. (2008). Improved survival in patients receiving medical therapy as compared with banding ligation for the prevention of esophageal variceal rebleeding. Hepatology.

[16] Bhutta A.Q., Garcia-Tsao G., Reddy K.R., Tandon P., Wong F., O’Leary J.G., Acharya C., Banerjee D., Abraldes J.G., Jones T.M. (2018). Beta-blockers in hospitalised patients with cirrhosis and ascites: Mortality and factors determining discontinuation and reinitiation. Aliment. Pharmacol. Ther..

[17] Sersté T., Melot C., Francoz C., Durand F., Rautou P.E., Valla D., Moreau R., Lebrec D. (2010). Deleterious efects of beta-blockers on survival in patients with cirrhosis and refractory ascites. Hepatology.

[18] Krag A., Wiest R., Albillos A., Gluud L.L. (2012). The window hypothesis: Haemodynamic and non-haemodynamic efects of β-blockers improve survival of patients with cirrhosis during a window in the disease. Gut.

[19] Hillon P., Lebrec D., Muńoz C., Jungers M., Goldfarb G., Benhamou J.P. (1982). Comparison of the effects of a cardioselective and a nonselective bblocker on portal hypertension in patients with cirrhosis. Hepatology.

[20] Westaby D., Melia W.M., Macdougall B.R., Hegarty J.E., Gimson A.E., Williams R. (1985). B1 selective adrenoreceptor blockade for the long-term management of variceal bleeding. A prospective randomised trial to compare oral metoprolol with injection sclerotherapy in cirrhosis. Gut.

[21] Torregrosa M., Genesca J., Gonzalez A., Evangelista A., Mora A., Margarit C., Esteban R., Guardia J. (2005). Role of Doppler echocardiography in the assessment of porto-pulmonary hypertension in liver transplantation candidates. J. Hepatol..

[22] López-Candales A., Menendez F.L., Shah S.A., Friedrich A. (2014). Measures of right ventricular systolic function in end stage liver disease patients awaiting transplant. Int. J. Cardiol..

[23] Chen Y., Chan A.C., Chan S.C., Chok S.H., Sharr W., Fung J., Liu J.H., Zhen Z., Sin W.C., Lo C.M. (2016). A detailed evaluation of cardiac function in cirrhotic patients and its alteration with or without liver transplantation. J. Cardiol..

[24] Sussman N.L., Kochar R., Fallon M.B. (2011). Pulmonary complications in cirrhosis. Curr. Opin. Organ. Transplant..

[25] Lang R.M., Badano L.P., Mor-Avi V., Afilalo J., Armstrong A., Ernande L., Flachskampf F.A., Foster E., Goldstein S.A., Kuznetsova T. (2015). Recommendations for cardiac chamber quantification by echocardiography in adults: An update from the American Society of Echocardiography and the European Association of Cardiovascular Imaging. J. Am. Soc. Echocardiogr..

[26] Rudski L.G., Lai W.W., Afilalo J., Hua L., Handschumacher M.D., Chandrasekaran K., Solomon S.D., Louie E.K., Schiller N.B. (2010). Guidelines for the echocardiographic assessment of the right heart in adults: A report from the American Society of Echocardiography endorsed by the European Association of Echocardiography, a registered branch of the European Society of Cardiology, and the Canadian Society of Echocardiography. J. Am. Soc. Echocardiogr..

[27] Haddad F., Doyle R., Murphy D.J., Hunt S.A. (2008). Right ventricular function in cardiovascular disease, partII: Pathophysiology, clinical importance, and management of right ventricular failure. Circulation.

[28] Voelkel N.F., Quaife R.A., Leinwand L.A., Barst R.J., McGoon M.D., Meldrum D.R., Dupuis J., Long C.S., Rubin L.J., Smart F.W. (2006). Right ventricular function and failure: Report of a National Heart, Lung, and Blood Institute working group on cellular and molecular mechanisms of right heart failure. Circulation.

[29] Matthews J.C., Dardas T.F., Dorsch M.P., Aaronson K.D. (2008). Right-sided heart failure: Diagnosis and treatment strategies. Curr. Treat. Opt. Cardiovasc. Med..

[30] Hernandez-Suarez D., López-Candales A. (2017). Subclinical Right Ventricular Dysfunction in Patients with Severe Aortic Stenosis: A Retrospective Case Series. Cardiol. Ther..

[31] Towheed A., Sabbagh E., Gupta R., Assiri S., Chowdhury M.A., Moukarbel G.V., Khuder S.A., Schwann T.A., Bonnell M.R., Cooper C.J. (2021). Right ventricular dysfunction and short-term outcomes following left-sided valvular surgery: An echocardiographic study. J. Am. Heart Assoc..

[32] Demirtaş Inci S., Sade L.E., Altın C., Pirat B., Erken Pamukcu H., Yılmaz S., Müderrisoğlu H. (2019). Subclinical myocardial dysfunction in liver transplant candidates determined using speckle tracking imaging. Turk. Kardiyol. Dern. Ars..

[33] Acosta F., Sansano T., Palenciano C.G., Roqués V., Clavel N., González P., Robles R., Bueno F.S., Ramirez P., Parrilla P. (2016). Relationship Between Cardiovascular State and Degree of Hepatic Dysfunction in Patients Treated with Liver Transplantation. Transplant Proc..

[34] Xu H., Li W., Xu Z., Shi X. (2012). Evaluation of the right ventricular ejection fraction during classic orthotopic liver transplantation without venovenous bypass. Clin. Transplant..

[35] Jeong Y.H., Yang S.-M., Cho H., Ju J.-W., Jang H.S., Lee H.-J., Kim W.H. (2021). The Prognostic Role of Right Ventricular Stroke Work Index during Liver Transplantation. J. Clin. Med..

[36] Gjeorgjievski M., Cappell M.S. (2016). Portal hypertensive gastropathy: A systematic review of the pathophysiology, clinical presentation, natural history and therapy. World J. Hepatol..

[37] Li T., Ke W., Sun P., Chen X., Belgaumkar A., Huang Y., Xian W., Li J., Zheng Q. (2016). Carvedilol for portal hypertension in cirrhosis: Systematic review with meta-analysis. BMJ Open..

[38] La Mura V., Abraldes J.G., Raffa S., Retto O., Berzigotti A., García-Pagán J.C., Bosch J. (2009). Prognostic value ofacute hemodynamic response to i.v. propranolol in patients with cirrhosis and portal hypertension. J. Hepatol..

[39] Garcia-Tsao G., Sanyal A.J., Grace N.D., Carey W., Practice Guidelines Committee of the American Association for the Study of Liver Diseases, Practice Parameters Committee of the American College of Gastroenterology (2007). Prevention and management of gastroesophageal varices and variceal hemorrhage in cirrhosis. Hepatology.

[40] Villanueva C., Albillos A., Genescà J., Garcia-Pagan J.C., Calleja J.L., Aracil C., Bañares R., Morillas R.M., Poca M., Peñas B. (2019). β blockers to prevent decompensation of cirrhosis in patients with clinically significant portal hypertension (PREDESCI): A randomised, double-blind, placebo-controlled, multicentre trial. Lancet.

[41] Brito-Azevedo A., Perez R.M., Coelho H.S., Fernandes E.S., Castiglione R.C., Villela-Nogueira C.A., Bouskela E. (2017). The anti-inflammatory role of propranolol in cirrhosis: Preventing the inflammatory exhaustion?. J. Hepatol..

[42] Reiberger T., Ferlitsch A., Payer B.A., Mandorfer M., Heinisch B.B., Hayden H., Lammert F., Trauner M., Peck-Radosavljevic M., Vogelsang H. (2013). Non-selective betablocker therapy decreases intestinal permeability and serum levels of LBP and IL-6 in patients with cirrhosis. J. Hepatol..

[43] Brito-Azevedo A., Perez Rde M., Coelho H.S., Fernandes Ede S., Castiglione R.C., Villela-Nogueira C.A., Bouskela E. (2016). Propranolol improves endothelial dysfunction in advanced cirrhosis: The ‘endothelial exhaustion’ hypothesis. Gut.

[44] Vilas-Boas W.W., Ribeiro-Oliveira A., Ribeiro Rda C., Vieira R.L., Almeida J., Nadu A.P., Simões e Silva A.C., Santos R.A. (2008). Effect of propranolol on the splanchnic and peripheral renin angiotensin system in cirrhotic patients. World J. Gastroenterol..

[45] Willett I., Esler M., Burke F., Leonard P., Dudley F. (1985). Total and renal sympathetic nervous system activity in alcoholic cirrhosis. J. Hepatol..

[46] Murray J.F., Dawson A.M., Sherlock S. (1958). Circulatory changes in chronic liverdisease. Am. J. Med..

[47] Rosoff L., Zia P., Reynolds T., Horton R. (1975). Studies of renin and aldosterone in cirrhotic patients with ascites. Gastroenterology.

[48] Rosoff L., Williams J., Moult P., Williams H., Sherlock S. (1979). Renal hemodynamics, and the renin–angiotensin system in cirrhosis: Relationship to sodium retention. Dig. Dis. Sci..

[49] de Franchis R., Bosch J., Garcia-Tsao G., Reiberger T., Ripoll C., Baveno VII Faculty (2022). Baveno VII-Renewing consensus in portal hypertension. J. Hepatol..

